# Application of Genome Editing Techniques in Immunology

**DOI:** 10.1007/s00005-018-0504-z

**Published:** 2018-01-17

**Authors:** Agata O. Zych, Malgorzata Bajor, Radoslaw Zagozdzon

**Affiliations:** 1 Department of Immunology, Medical University of Warsaw, Warsaw, Poland; 2 Postgraduate School of Molecular Medicine, Medical University of Warsaw, Warsaw, Poland; 3 Department of Immunology, Transplantology and Internal Medicine, Medical University of Warsaw, Warsaw, Poland; 4 Institute of Biochemistry and Biophysics, Polish Academy of Sciences, Warsaw, Poland; 5 Department of Clinical Immunology, Medical University of Warsaw, Nowogrodzka 59, 02-006 Warsaw, Poland

**Keywords:** Adoptive therapy, Cancer, Viral diseases, Immunotherapy, TALEN, CRISPR/Cas9, Genome editing

## Abstract

The idea of using the effector immune cells to specifically fight cancer has recently evolved into an exciting concept of adoptive cell therapies. Indeed, genetically engineered T cells expressing on their surface recombinant, cancer-targeted receptors have been shown to induce promising response in oncological patients. However, in addition to exogenous expression of such receptors, there is also a need for disruption of certain genes in the immune cells to achieve more potent disease-targeted actions, to produce universal chimeric antigen receptor-based therapies or to study the signaling pathways in detail. In this review, we present novel genetic engineering methods, mainly TALEN and CRISPR/Cas9 systems, that can be used for such purposes. These unique techniques may contribute to creating more successful immune therapies against cancer or prospectively other diseases as well.

## Introduction

The fundamental concept of adoptive cell therapies (ACT) against cancer or viral diseases is that immune effector cells can be isolated, expanded and returned to the patient to achieve a potent and disease-targeted cytotoxic activity (reviewed in Perica et al. 2015). For decades, however, ACT have been bringing only a modest success, as the classical recognition of target cells via endogenous T-cell receptor (TCR) is often inefficient for the cure (Dudley et al. 2008). This situation has been dramatically changed following introduction of genetic modifications of the effector cells that redirect them to target a chosen antigen (Fujiwara 2014). These modifications are usually following one out of two main streams: (1) introduction of recombinant α and β chains pairing into exogenous tumor-specific TCR or (2) introduction of a chimeric antigen receptor (CAR) targeting a chosen surface molecule on cancer cells.

Although much improved, the immune effector cells expressing exogenous cancer-specific receptors still face considerable limitations, mainly due to three types of factors. The first is their susceptibility to inhibition via the natural immune checkpoint signaling, e.g. the programmed death-1 (PD-1)-mediated route (John et al. 2013). The second factor is related to the presence of the endogenous TCR, that following activation of the effector cell can potentially mediate severe autoimmune complications of the autologous T-cell transplant or graft-versus-host disease in allogeneic settings. Also in this context, the heterologous pairing of the α and β chains of recombinant TCR with respective chains of endogenous TCR chains may attenuate their antigenic specificity or lead to autoreactivity (Heemskerk et al. 2007; van Loenen et al. 2010). Finally, the presence of intrinsic MHC class I molecules on the effector cells prevents their application in allogeneic settings as the off-the-shelf ACT, which makes the adoptive therapies considerably more expensive. To overcome these limitations, genome editing methods have been recently employed (Provasi et al. 2012).

Targeted genome editing (reviewed in Guha et al. 2017) constitutes a powerful tool for biological research and potential approach for genetic therapy. The most general concept behind genome editing is the introduction of double-strand breaks within DNA sequence in a region of interest, followed by an action of endogenous repair machinery to induce targeted mutations. The changes in the DNA structure can be repaired by two broad mechanisms: error-prone non-homologous end joining (NHEJ) or homology directed repair (HDR). In case of lack of a homologous repair template, the NHEJ may lead to insertion/deletion (in/dels) events, and thus cause changes in the open reading frame of the target gene (Martins-Rocha et al. 2015).

Lately, the most commonly used tools in genetic engineering are meganucleases (MN), zinc-finger nucleases (ZFN), transcription activator-like effector nucleases (TALEN) and clustered regularly interspaced short palindromic repeats (CRISPR) complexes, with the substantial predominance of the last technique in recent years. The main advantages and shortcomings of gene editing methods are summarized in Table 1.

**Table 1:** Comparison of the main genome editing methods

Method	Advantages	Disadvantages	Limitations
MN	High specificity Low toxicity Recognition of large DNA sequences	Extremely laborious Single domain encoding two important MN functions: recognition and cleavage	Insufficient variety of recognized sequences
ZFN	Recognition of any sequence High efficiency	High cost Complexity of protein domains Pairs of ZFNs are required to target any specific locus Requires screening to detect targeted events in animals Off-target effects	ZFN recognizes 3–6 nucleotide sequences
TALEN	TALE monomer recognizes single nucleotide in target sequence Lower cost than ZFN High specificity	Identical repeat sequences within TALE array—cloning challenge Complexity of protein domains Large size of TALE molecules difficult to deliver to the cells Pairs of TALENs are required to target any specific locus	Binding efficiency depends on the presence of thymidine nucleotide before the 5′ end of a sequence
CRISPR/Cas9	Simplicity Efficiency Low cost High precision Versatility Multiplexed mutations Ability to obtain mutant organism in one generation	High possibility of off-target effects Mosaicism	Limited target sequences due to necessity of presence of PAM sequences

*MN* meganucleases, *ZFN* zinc-finger nucleases, *TALEN* transcription activator-like effector nucleases, *CRISPR* clustered regularly interspaced short palindromic repeats, *PAM* proto-spacer-adjacent motifs

## Meganucleases

Meganucleases, also called homing endonucleases, are an engineered version of naturally occurring endonucleases, which are able to recognize and cleave considerably large DNA sequences (~ 14–40 bps) very rare in the most genomes (Stoddard 2011). Recognition of the unique sequences makes MN a very specific, non-toxic and highly suitable tool for genome engineering. However, the insufficiency of naturally occurring MN and limited variety of recognized sequences constitute the main drawbacks of this method. Moreover, the recognition and cleavage functions of MN are encoded in a single domain where the part of their structure is involved in a complex system of DNA interactions. The intricacy of the desired targeted sequence design has been partially solved by few scientific groups using fusion chimeras or mutating specific residues in the DNA binding scaffold (Silva et al. 2011; Zaslavskiy et al. 2014). Additionally, various companies managed to develop procedures to modify MN for use in genome editing to induce targeted recombination and correction of the RAG1 gene related to severe combined immunodeficiency (SCID) (Grizot et al. 2009) or XPC gene associated with xeroderma pigmentosum in skin cells (Arnould et al. 2007). A recently published study has shown a successful application of meganuclease-mediated TCR α-chain knock-out under conditions for optimal T-cell stimulation (MacLeod et al. 2017). Nonetheless, the procedure of “programming” MN to recognize the given sequence requires specialized knowledge and technology, and makes this approach extremely laborious. Due to the fact that meganucleases are very difficult to optimize to target specific sequence, MN have not been widely used for genome engineering.

## Zinc-Finger Nucleases

ZFN are fusion proteins with engineered DNA binding domains and a non-specific nuclease domain from the *Fok*I restriction enzyme. ZFN were the first reagents utilized to introduce targeted changes into the genome (Durai et al. 2005). Individual ZF motif consists of approximately 30 amino acids organized in a conserved ββα structure stabilized by the hydrophobic cluster of residues and chelation of the zinc ion. The DNA binding is performed by interaction of several amino acids of the ZF α-helix with three base pairs in the major groove of DNA (Gaj et al. 2013). Typically, each ZFN recognizes 3–6-nucleotide sequences. ZF motifs can be designed to recognize almost any DNA sequence. Nucleases attached to ZF work as dimers, thus pairs of ZFN are required to target any specific locus (Durai et al. 2005). Despite a theoretical possibility to target any specific sequence, ZFN approach has in fact a number of major disadvantages. Primarily, the high cost and complexity of protein domains design make this method unattractive. Furthermore, single nucleotide substitutions or improper interactions between domains increase the probability of inaccurate cleavage of target sequence (Nemudryi et al. 2014). Nevertheless, ZFN can potentially be of use for editing the genome of T cells mainly in HIV-related research (Perez et al. 2008), especially when combined with adeno-associated virus vectors to function as homology donors (Wang et al. 2016). ZFN approach was also used to mediate site-specific integration of therapeutic transgenes in hepatocytes within albumin gene. Expression of human factors VIII and IX were obtained in mouse models of hemophilia A and B at therapeutic levels as well as lysosomal enzymes that are deficient in Fabry and Gaucher diseases and in Hurler and Hunter syndromes (Sharma et al. 2015). What is more, ZFN technology have been successfully used to disrupt *CCR5* gene in hematopoietic stem/progenitor cells (HSPC) (DiGiusto et al. 2016). Currently there are several ongoing clinical studies utilizing ZFN approach against HIV-1 infection, hemophilia B or mucopolysaccharidosis I/II (see Table 2).

**Table 2:** Examples of clinical trials incorporating gene-editing methods into treatment of various human diseases

Trial identifier	Technology	Target	Study population	Status	Study phase	Country
NCT02500849	ZFN	Disruption of a *CCR5* gene in HSPCs	HIV-1 infected patients	Ongoing	Phase I	USA
NCT02800369	ZFN	Disruption of *HPV16* and *HPV18 E7* oncogenes specifically in cervical precancerous lesions	Female patients with documented HPV16 or HPV18 infection	Ongoing	Phase I	China
NCT00842634	ZFN	Disruption of a *CCR5* gene in T cells	HIV-1 infected patients	Completed	Phase I	USA
NCT01252641	ZFN	Disruption of a *CCR5* gene in T cells	HIV-1 infected patients	Completed	Phase I/II	USA
NCT02388594	ZFN	Disruption of a *CCR5* gene in T cells	HIV-1 infected patients	Ongoing	Phase I	USA
NCT02225665	ZFN	Disruption of a *CCR5* gene in T cells	HIV-1 infected patients	Ongoing	Phase I/II	USA
NCT01543152	ZFN	Disruption of a *CCR5* gene in T cells	HIV-1 infected patients	Ongoing	Phase I/II	USA and Puerto Rico
NCT02695160	ZFN	Insertion of *Factor 9* gene into the albumin locus in hepatocytes	Patients with hemophilia B	Ongoing	Phase I	USA
NCT02702115	ZFN	Insertion of a correct copy of the *IDUA* gene into the Albumin locus in hepatocytes	Patients with attenuated MPS I deficiency	Ongoing	Phase I	USA
NCT03041324	ZFN	Insertion of a correct copy of the *IDS* gene into the albumin locus in hepatocytes	Patients with attenuated MPS II deficiency	Ongoing	Phase I	USA
NCT03057912	TALEN/CRISPR/Cas9	Disruption of a *HPV16 and HPV18 E6*/*E7* oncogenes	Females with documented HPV16 or HPV18 infection	Begining in 2018	Phase I	China
NCT03226470	TALEN	Disruption of a *HPV16 E6 and E7* oncogenes	Females with documented HPV16 or HPV18 infection	Begining in 2018	Phase I	China
NCT03164135	CRISPR/Cas9	Disruption of a *CCR5* gene in CD34^+^ HSPCs	HIV-infected patients with hematological malignances	Ongoing	Not provided	China
NCT03166878	CRISPR/Cas9	Introduction of CAR T cells against CD19 on B cells	Patients with relapsed or refractory CD19 positive B-cell leukemia or lymphoma	Ongoing	Phase I/ II	China
NCT03081715	CRISPR/Cas9	Disruption of a *PDCD-1* gene in T cells	Patients with histologically confirmed recurrent or metastatic esophageal cancer	Ongoing	Phase II	China
NCT02863913	CRISPR/Cas9	Disruption of a *PDCD-1* gene in T cells	Patients with stage IV muscle-invasive bladder cancer with measurable lesions	Ongoing	Phase I	China
NCT02867332	CRISPR/Cas9	Disruption of a *PDCD-1* gene in T cells	Patients with stage IV renal cancer with measurable lesions	Ongoing	Phase I	China
NCT02867345	CRISPR/Cas9	Disruption of a *PDCD-1* gene in T cells	Patients with pathologically and clinical verified castration resistant prostate cancer with measurable lesions	Ongoing	Phase I	China
NCT02793856	CRISPR/Cas9	Disruption of a *PDCD-1* gene in T cells	Patients with pathologically verified stage IV non-small cell lung cancer with measurable lesions	Ongoing	Phase I	China
NCT03044743	CRISPR/Cas9	Disruption of a *PDCD-1* gene in EBV-specific cytotoxic T cells	Patients with pathologically verified stage IV gastric carcinoma, nasopharyngeal carcinoma and lymphoma with measurable lesions Pathologically verified as EBV positive malignancies	Ongoing	Phase I/II	China

*HPV* human papillomavirus, *EBV* Epstein–Barr virus, *MPS I* mucopolysaccharidosis I, *IDUA* α-l-iduronidase

## Transcription Activator-Like Effector Nucleases

**Fig. 1:**
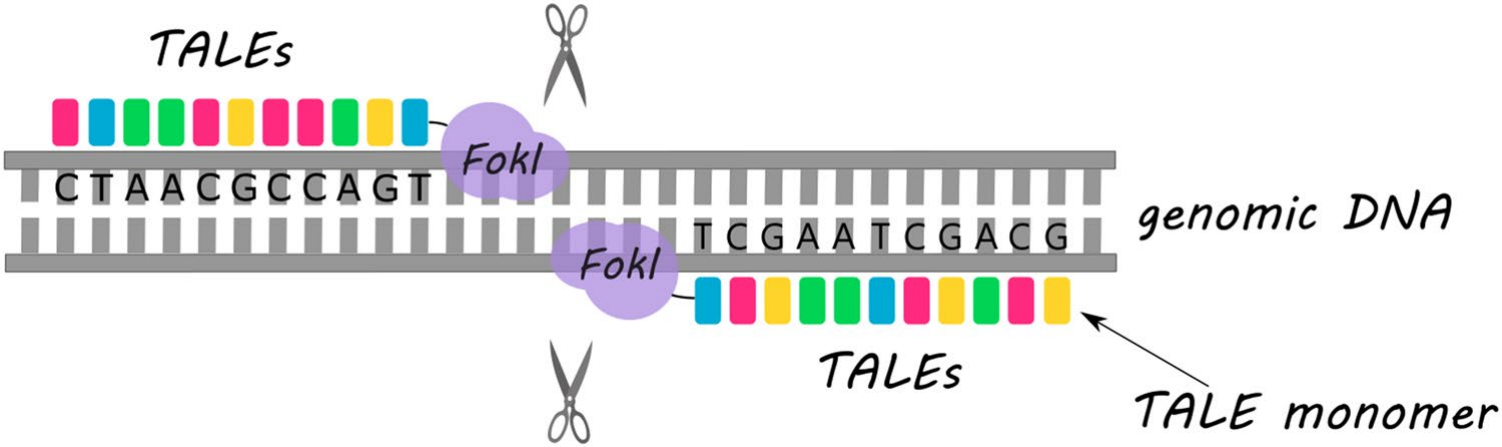
Schematic representation of the double-strand DNA break introduction using TALEN. *Fok*I enzyme acts as a catalytic domain following the recognition of specific DNA sequences by TALEs (depicted as colorful rectangles)

The method that was considered to overcome the ZFN drawbacks was TALEN (Fig. 1). Similarly to ZFN, the DNA binding domain that is fused with *Fok*I enzyme in TALEN structure consists of a sequence of protein monomers (called TALE). Unlike ZFN, a single TALE monomer binds to one nucleotide in the target sequence. The ability of TALEN method to recognize single bases is an unquestionable advantage in targeting desirable sequence in contrast to ZFN approach which recognizes nucleotide triplets. Each TALE monomer is composed of a series of 33–35 amino acid repeat domains. The two highly variable amino acid residues located at positions 12 and 13 (called repeat variable diresidue) are responsible for TALE specificity (Gaj et al. 2013; Joung and Sander 2013; Nemudryi et al. 2014). In TALEN, TALE monomers can be arbitrarily linked together to recognize the desired DNA sequence. However, due to the expanded identical repeat sequences, cloning of TALE arrays causes a major technical challenge (Christian et al. 2010; Miller et al. 2011). Furthermore, the critical point for binding efficiency is the presence of thymidine nucleotide before the 5′ end of a sequence bound by TALE monomer (Lamb et al. 2013).

TALEN technology has been utilized in ACT strategies on numerous occasions. For instance, Poirot et al. (2015) have described the TALEN-mediated multiplex genome-edited manufacturing platform for universal T-cell-based immunotherapies. Based on a similar approach, a successful application of the TALEN-edited [by disruption of TCRα constant (*TRAC*) and *CD52* genes] CAR-T cells targeting CD19 in two HLA-mismatched infants with relapsed refractory B-cell acute lymphoblastic leukemia has been recently reported (Qasim et al. 2017). TALEN strategy has also been used to inactivate the PD-1 molecule in tumor-reactive lymphocytes (Menger et al. 2016). Two upcoming clinical trials are going to use this methodology in treatment of female patients with human papillomavirus (HPV)-related cervical intraepithelial neoplasia (see Table 2).

## CRISPR/Cas9

Elucidation of the role of identified clustered regularly interspaced short palindromic repeats found for the first time in *Escherichia coli* in 1987 (Ishino et al. 1987) have revolutionized the manipulation of DNA and introduction of site-specific mutations. CRISPR/Cas is an adaptive immune system (reviewed in Hryhorowicz et al. 2017) found in many bacteria and archaea, which enables effective defense against the invasion of bacteriophages or viruses. This immune system allows prokaryotes to “memorize” foreign DNA by incorporating its fragments into CRISPR arrays and ensures fast response to another infection in the future (Barrangou et al. 2007). The CRISPR array is organized by series of short (approx. 23–44 bp) sequences called spacers which are separated by highly conserved similarly sized sequences repeats. These spacers originate from viral or phage DNA and serve as a genetic memory of previous infections (Barrangou et al. 2007; Bolotin et al. 2005; Garneau et al. 2010; van der Ploeg 2009). Another very important compound of this system are Cas (CRISPR associated proteins) endonucleases, which mediate the double-strand breaks.

The CRISPR/Cas immune system performs its function in three general steps: adaptation, expression, and interference. During the first stage, short fragments of viral or phage DNA are incorporated into the CRISPR array. The integration of the new viral/phage DNA sequences is followed by duplication of a repeat, which in this way is forming a new spacer-repeat unit. Spacer precursors called proto-spacers are selected from invading DNA depending on the recognition of neighboring proto-spacer-adjacent motifs (PAM). PAM sequences are typically several nucleotides long and vary among different variants of the CRISPR/Cas system (Makarova et al. 2011). The arrangement of spacers within the CRISPR array corresponds to the sequence of invasion events. In the next stage, CRISPR array is transcribed and primary transcript pre-crRNA is produced, which then is processed to mature CRISPR RNA (crRNA) by RNase III. Depending on the CRISPR/Cas system class, this process can be mediated either by multiprotein CRISPR ribonucleoprotein complex or a single protein. In the last step—interference, crRNA directs Cas proteins to appropriate target within foreign DNA or RNA and Cas proteins perform cleavage of the invading genome (Terns and Terns 2013).

**Fig. 2:**
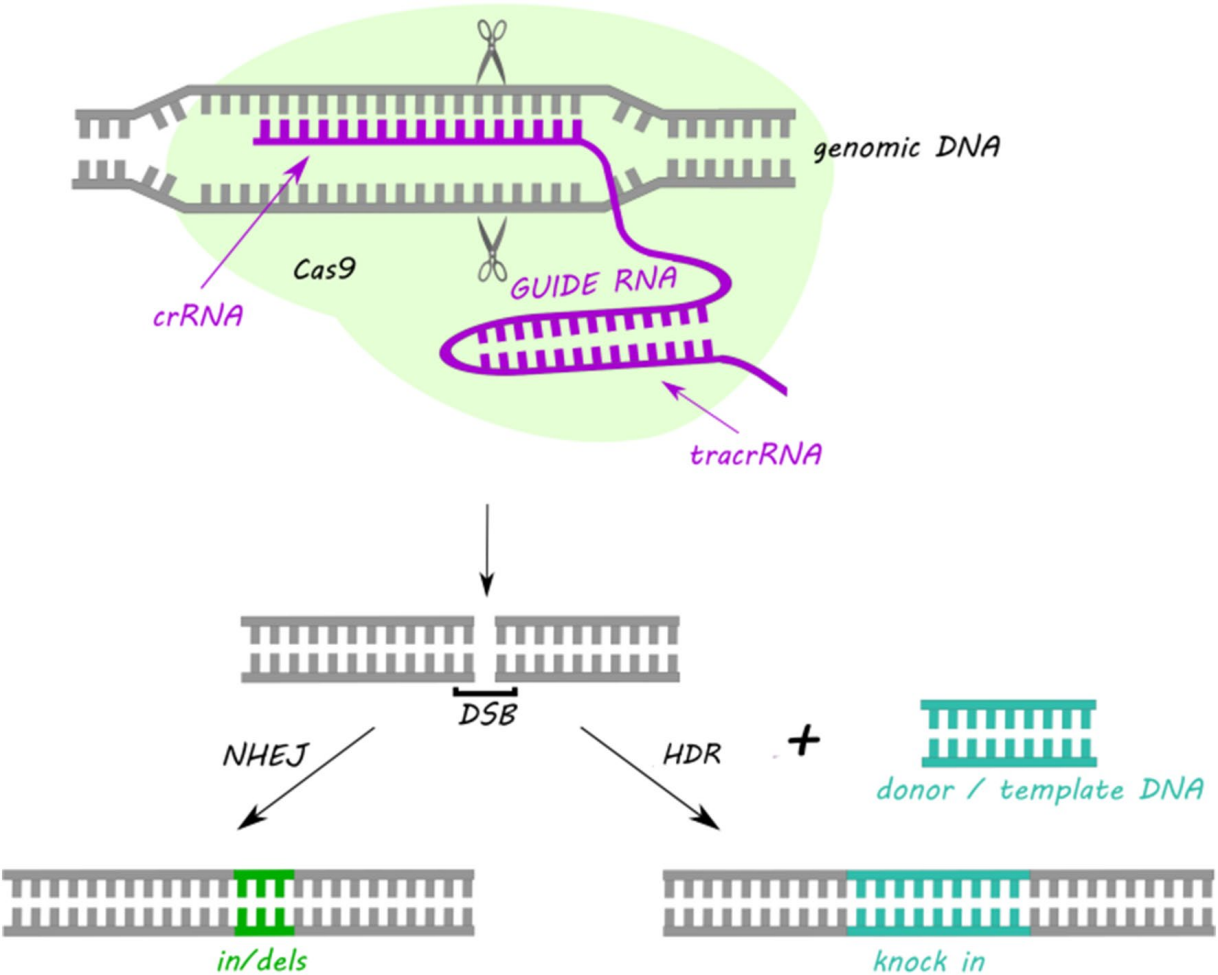
Schematic representation of the double-strand DNA break introduction by a sgRNA-guided CRISPR/Cas9-based system and the main routes of DNA repairing. *NHEJ* non-homologous end joining, *DSB* double-strand breaks, *HDR* homology directed repair

Despite the variety of the CRISPR/Cas systems in nature, the most commonly used type adapted to genome editing is class 2 type II CRISPR/Cas9. CRISPR/Cas9 requires two short RNA sequences: crRNA and transactivating crRNA (tracrRNA) to recognize and cleave foreign DNA sequences. During the action of Cas9 the crRNA hybridizes with the tracrRNA forming duplex crRNA:tracrRNA, which in the next step associates with Cas9. The crRNA is complementary to the target DNA sequence, while tracrRNA shows homology towards PAM and possess a binding site for the Cas9 which is indispensable for interference step (Karvelis et al. 2013). The Cas9 comprises of two nuclease domains: HNH responsible for cleavage of the DNA strand complementary to the spacer sequence and RuvC that cleaves noncomplementary strand (Nishimasu et al. 2014). The most frequently commercially utilized version of CRISPR/Cas9 system consists of Cas9 protein from *Streptococcus pyogenes* and a chimeric single guide RNA (sgRNA), that is a fusion of crRNA and tracrRNA (Fig. 2). sgRNA can be designed to target any sequence followed by a 5′-NGG-3′ PAM sequence (Cong et al. 2013; Mali et al. 2013). Moreover, multiple genes can be targeted at the same time by introducing multiple sgRNAs at once (Cong et al. 2013). Despite the high efficiency, feasibility, and simplicity of target design CRISPR/Cas9 technique faces important complications. The most essential limitation of this method are mutations at sites with similar but not identical homology to the target sites (Cradick et al. 2013). To overcome random mutations, a number of modifications have been introduced to CRISPR/Cas9 strategy, as described below.

One possibility involves shortening of sgRNA to create truncated sgRNA (trugRNA) to the length of less than 20 nucleotides (17–19) and this manipulation decreases undesired mutagenesis by 5000-fold without compromising the efficiency (Fu et al. 2014). The other option is to convert Cas9 nucleases into nickases that enhance genome editing specificity. Cas9 nickases possess mutation in one of the endonuclease domains (RuvC^D10A^ or HNH^H840A^) and hence cut only one strand of DNA generating single-strand breaks. Repair of individual nicks in the genome occur with high fidelity, without inducing in/dels, therefore, introduction of paired nicking can reduce unwanted off-target activity by 50–1000-fold (Chiang et al. 2016; Ran et al. 2013; Shen et al. 2014). Efficient reduction of in/dels caused by NHEJ mechanism was achieved also by inhibiting DNA ligase IV, a key enzyme in NHEJ pathway. This alteration showed great improvement in the efficiency of precise editing by CRISPR/Cas9 in fertilized zygotes and may be applicable also in other genetic engineering methods such as ZFN or TALEN (Maruyama et al. 2015).

Another way to reduce the occurrence of off-target mutations is generation of dimeric RNA-guided *Fok*I nucleases (RFNs), that are able to recognize extended sequences and introduce modifications with high efficiencies. RFNs are created by fusing, wild-type *Fok*I nuclease domain to catalytically inactive Cas9 (dCas9) protein. The *Fok*I nuclease domain requires dimerization to perform DNA cleavage. Thus, it is highly unlikely that any mutagenesis could be introduced at partially mismatched, off-target half-sites. Indeed, no detectable mutations were found in known off-target sites within targeted sequences with the use of RFN approach (Tsai et al. 2014). dCas9 alone enables targeting genomic DNA without cleaving it as a flexible and precise RNA-guided transcription regulation. This ability of dCas9 was applied in Cas9-SunTag system. SunTag is a repeating peptide array, which can recruit multiple copies of an antibody-fusion protein. In dCas9-SunTag were employed multiple VP64 domains (VP64—four copies of herpes virus transcriptional activation domain VP16) to a single dCas9 and this manipulation enhanced potently artificial activation of gene transcription without introducing genetic changes (Tanenbaum et al. 2014).

Apart from gene disruption and transcription activation, the CRISPR/Cas9 method enables introduction of a gene knock-in as well. The methodology is quite similar to gene knock-out and requires sgRNA, which targets the knock-in site, Cas9 protein and additionally a donor sequence of interest. In primary T cells, Cas9 ribonucleoproteins were used for targeted nucleotide replacement *CXCR4* and *PDCD1* genes (Schumann et al. 2015).

With the emergence of CRISPR/Cas9 method, the modifications of hematopoietic progenitors cells or T cells have become easier to achieve. Indeed, numerous of such attempts have been made (Chi et al. 2016; Chu et al. 2016; Gundry et al. 2016; Gwiazda et al. 2016; Hendel et al. 2015; Li et al. 2015; Liu et al. 2017; Mandal et al. 2014; Schumann et al. 2015, Su et al. 2016, 2017), as exemplified in Table 3. Various clinically important genes were disrupted in human primary CD4^+^ T cells and/or CD34^+^ hematopoietic and progenitor cells with high on-target efficacy and low incidence of off-target mutagenesis using CRISPR/Cas9 approaches e.g.: β_2_-microglobulin (B2M)—encoding the accessory chain of major histocompatibility complex (MHC) class I (Liu et al. 2017; Mandal et al. 2014); chemokine receptor 5 (CCR5)—the main coreceptor used by CCR5-tropic HIV-1 strains (Mandal et al. 2014; Hendel et al. 2015; Li et al. 2015; Gwiazda et al. 2016); IL2RG—encoding common gamma chain of many interleukin receptors which mutations are responsible for SCID-X_1_, HBB—encoding β-globin, mutations within this gene cause sickle anemia and thalassemia (Hendel et al. 2015); CXCR4—an α chemokine receptor, used by HIV virus to infect T cells (Schumann et al. 2015) and PDCD1, encoding PD-1 (Schumann et al. 2015, Su et al. 2016, 2017; Liu et al. 2017). Yet, the greatest interest is focused around introduction of CAR-T cells. The concept of making an adoptive immunotherapy accessible for every patient, even those without enough good quality T cells, is to develop an allogeneic adoptive transfer. This idea assumes creating universal CAR-T cells obtained from a healthy donor T cells and further application of modified T cells to multiple patients. As mentioned above, to succeed the endogenous αβ TCR need to be disrupted as well as MHC class I to minimize their immunogenicity. Recent publication is showing a great potential of modified T cells by CRISPR/Cas9 in this context. Anti-CD19 CAR has already been reported to possess a potent anti-tumor activity in B-cell non-Hodgkin lymphoma, acute lymphoblastic leukemia or chronic lymphocytic leukemia patients (Turtle et al. 2016). Liu et al. (2017) obtained double (B2M, TRAC) or triple knock-outs (B2M, TRAC and PD-1) with the use of CRISPR/Cas9 technology and insertion of anti-CD19 CAR in T cells. All variants were tested for off-target mutations and no significant differences between control cells and knock-out/knock-in cells were found. Double knock-out cells showed reduced proliferation as a result of loss of TCR function. Importantly, the cytotoxic functions remained unchanged in comparison to the standard CAR-T cells. Furthermore, in vivo experiment showed a maintained CD19 anti-tumor specificity despite multiplex gene editing with CRISPR/Cas9 (Liu et al. 2017). CRISPR/Cas9 method shows great potential for genome editing even in modifications of T cells. Apart from the therapeutic use, CRISPR/Cas9 can also be utilized for studying the intracellular signaling in T cells (Chi et al. 2016).

**Table 3:** Examples of genetic modifications of T lymphocytes or hematopoietic progenitor cells with the use of CRISPR/Cas9-based methods

Modification/s	Targeted molecules	Cell type	References
Gene disruption	B2M; CCR5	Human primary CD4^+^ T cells CD34^+^ hematopoietic progenitor cells	Mandal et al. (2014)
Gene disruption	IL2RG, HBB and CCR5	Human primary cells	Hendel et al. (2015)
Gene disruption Knock-in	CXCR4, PD1 12 nucleotides (of CXCR4 or PD-1 gene) with PAM sequence and restriction site for Hind III	Human primary CD4^+^ T cells	Schumann et al. (2015)
Gene disruption	CCR5	Human primary CD4^+^ T cells	Li et al. (2015)
Gene disruption	PD-1	Human primary T cells	Su et al. (2016)
Gene disruption	Genes encoding B-cell or T-cell surface markers (Rosa26, Prdm1, Ost4, Arf4, Creld2, Zfp36, Edem1, Irf4, Myc, Xbp1, Pou2af1)	Murine T cells and B cells	Chu et al. (2016)
Gene disruption	Eed, Suz12, and DNMT3A	Human and mouse hematopoietic progenitor cells	Gundry et al. (2016)
Gene disruption Upregulation of expression	CD28 CXCR4	JK28 cells JS19	Chi et al. (2016)
Gene disruption	TCRα, TIGIT, Lag3, Tim3 and CCR5	Human primary T cells	Gwiazda et al. (2016)
Gene disruption	PD-1	T cells	Su et al. (2017)
Gene disruption Knock-in	TRAC, B2M, PD-1 Anti-CD19 CAR	T cells	Liu et al. (2017)

In clinical settings, researchers in China have recently initiated a clinical trial to evaluate CAR-T cells modified by the CRISPR/Cas9 method (ClinicalTrials.gov Identifier: NCT03166878). They will combine the lentiviral delivery of anti-CD19 CAR and CRISPR RNA electroporation to disrupt endogenous TCR- and B2M-encoding genes simultaneously. Several other Chinese clinical trials are investigating the efficacy of CRISPR/Cas9-mediated PD-1 knock-out T cells in patients with malignancies such as: advanced esophageal carcinoma (NCT03081715), muscle-invasive bladder cancer (NCT02863913), metastatic renal cell carcinoma (NCT02867332) and others (see Table 2).

## Prospects of Gene Editing Methods in Treatment of Diseases

Gene therapy holds promise of an attractive and expectantly precise treatment of variety of diseases in the future. Enabling treating illnesses caused by gene mutations by replacing DNA fragments with a correct copy of the gene or inactivating improperly functioning genes are widely studied subjects. Despite various drawbacks and limitations of gene editing methods (see Table 1), they have already been successfully used in a number of clinical studies (Table 2). These trials often reach beyond cancer treatment. For instance, several clinical studies are utilizing ZFN technology to disrupt *CCR5* gene in HSPC or T cells which is required for HIV virus to enter into T cells (NCT02500849; NCT02388594; NCT02225665; NCT01543152). A number of similar clinical trials conducted earlier, have revealed that ZFN approach is mostly safe for application in humans (NCT00842634, NCT01044654). Generally, infusion of ZFN-modified autologous T cells was associated with mild side effects and only one serious side effect was observed in relation to transfusion. During those studies, a significant increase of CD4^+^ T cells was observed. What is more, HIV DNA decreased in most patients and HIV RNA was undetectable in one of four evaluated patients (Tebas et al. 2014). Those results give an encouraging starting point for the application of genetic engineering methods in treating various viral infections hampering functionality of the immune system, though this approach needs to be assessed in a wider group of patients. However, the complexity of ZFN and TALEN design may lead to more extensive development of simpler and more feasible ways to use CRISPR/Cas9 method. Furthermore, safety issues and off-target effects are being solved by various modifications such as: Cas9 nickase, using Cas9 mRNA or adeno-associated vectors for introduction of system components into the cells with high efficiency and little or no risk for the patient. The interesting example is given by a recent publication of Yin et al. (2016), where researchers utilizing CRISPR/Cas9 system successfully restored the correct *FAH* gene function in 6% of liver cells in a mouse model of tyrosinemia type I, which was enough to cure the disease. Thus, with emergence of easy, inexpensive and highly efficient CRISPR/Cas9-based methodology, more and more clinical trials are testing safety of this approach in treating various cancers or viral infections (see Table 3). One must assume that in the near future additional genome editing-based therapies will be available to treat various somatic diseases. Obviously, appearance of gene editing methods creates a temptation to therapeutically modify human embryos, however, discussion of these strategies ranges beyond the scope of the current review.

## Conclusion

In the last several years, we have observed a revolution in ACT used in oncology due to the capabilities of new methods for retargeting the immune effector cells against the cancer cells. Most recently, it has been increasingly clear that the gene editing techniques, such as TALEN or CRISPR/Cas9, may further refine ACT or direct genetic therapies to become a successful, universal and cost-effective strategy against cancer and perhaps a range of other diseases as well.

## References

[CR1] Arnould S, Perez C, Cabaniols JP (2007). Engineered I-CreI derivatives cleaving sequences from the human XPC gene can induce highly efficient gene correction in mammalian cells. J Mol Biol.

[CR2] Barrangou R, Fremaux C, Deveau H (2007). CRISPR provides acquired resistance against viruses in prokaryotes. Science.

[CR3] Bolotin A, Quinquis B, Sorokin A (2005). Clustered regularly interspaced short palindrome repeats (CRISPRs) have spacers of extrachromosomal origin. Microbiology.

[CR4] Chi S, Weiss A, Wang H (2016). A CRISPR-based toolbox for studying T cell signal transduction. Biomed Res Int.

[CR5] Chiang TW, le Sage C, Larrieu D (2016). CRISPR–Cas9D10A nickase-based genotypic and phenotypic screening to enhance genome editing. Sci Rep.

[CR6] Christian M, Cermak T, Doyle EL (2010). Targeting DNA double-strand breaks with TAL effector nucleases. Genetics.

[CR7] Chu VT, Graf R, Wirtz T (2016). Efficient CRISPR-mediated mutagenesis in primary immune cells using CrispRGold and a C57BL/6 Cas9 transgenic mouse line. Proc Natl Acad Sci USA.

[CR8] Cong L, Ran FA, Cox D (2013). Multiplex genome engineering using CRISPR/Cas systems. Science.

[CR9] Cradick TJ, Fine EJ, Antico CJ (2013). CRISPR/Cas9 systems targeting β-globin and CCR5 genes have substantial off-target activity. Nucleic Acids Res.

[CR10] DiGiusto DL, Cannon PM, Holmes MC (2016). Preclinical development and qualification of ZFN-mediated CCR5 disruption in human hematopoietic stem/progenitor cells. Mol Ther Methods Clin Dev.

[CR11] Dudley ME, Yang JC, Sherry R (2008). Adoptive cell therapy for patients with metastatic melanoma: evaluation of intensive myeloablative chemoradiation preparative regimens. J Clin Oncol.

[CR12] Durai S, Mani M, Kandavelou K (2005). Zinc finger nucleases: custom-designed molecular scissors for genome engineering of plant and mammalian cells. Nucleic Acids Res.

[CR13] Fu Y, Sander JD, Reyon D (2014). Improving CRISPR–Cas nuclease specificity using truncated guide RNAs. Nat Biotechnol.

[CR14] Fujiwara H (2014). Adoptive immunotherapy for hematological malignancies using T cells gene-modified to express tumor antigen-specific receptors. Pharmaceuticals.

[CR15] Gaj T, Gersbach CA, Barbas CF (2013). ZFN, TALEN and CRISPR/Cas-based methods for genome engineering. Trends Biotechnol.

[CR16] Garneau JE, Dupuis M, Villion M (2010). The CRISPR/Cas bacterial immune system cleaves bacteriophage and plasmid DNA. Nature.

[CR17] Grizot S, Smith J, Daboussi F (2009). Efficient targeting of a SCID gene by an engineered single-chain homing endonuclease. Nucleic Acids Res.

[CR18] Guha TK, Wai A, Hausner G (2017). Programmable genome editing tools and their regulation for efficient genome engineering. Comput Struct Biotechnol J.

[CR19] Gundry MC, Brunetti L, Lin A (2016). Highly efficient genome editing of murine and human hematopoietic progenitor cells by CRISPR/Cas9. Cell Rep.

[CR20] Gwiazda KS, Grier AE, Sahni J (2016). High efficiency CRISPR/Cas9-mediated gene editing in primary human T-cells using mutant adenoviral E4orf6/E1b55k “helper” proteins. Mol Ther.

[CR21] Heemskerk MH, Hagedoorn RS, Hoorn MA (2007). Efficiency of T-cell receptor expression in dual-specific T cells is controlled by the intrinsic qualities of the TCR chains within the TCR-CD3 complex. Blood.

[CR22] Hendel A, Bak RO, Clark JT (2015). Chemically modified guide RNAs enhance CRISPR–Cas genome editing in human primary cells. Nat Biotechnol.

[CR23] Hryhorowicz M, Lipiński D, Zeyland J (2017). CRISPR/Cas9 immune system as a tool for genome engineering. Arch Immunol Ther Exp.

[CR24] Ishino Y, Shinagawa H, Makino K (1987). Nucleotide sequence of the iap gene, responsible for alkaline phosphatase isozyme conversion in *Escherichia coli*, and identification of the gene product. J Bacteriol.

[CR25] John LB, Devaud C, Duong CP (2013). Anti-PD-1 antibody therapy potently enhances the eradication of established tumors by gene-modified T cells. Clin Cancer Res.

[CR26] Joung JK, Sander JD (2013). TALENs: a widely applicable technology for targeted genome editing. Nat Rev Mol Cell Biol.

[CR27] Karvelis T, Gasiunas G, Miksys A (2013). crRNA and tracrRNA guide Cas9-mediated DNA interference in *Streptococcus thermophilus*. RNA Biol.

[CR28] Lamb BM, Mercer AC, Barbas CF (2013). Directed evolution of the TALE N-terminal domain for recognition of all 5′ bases. Nucleic Acids Res.

[CR29] Li C, Guan X, Du T (2015). Inhibition of HIV-1 infection of primary CD4 + T-cells by gene editing of CCR5 using adenovirus-delivered CRISPR/Cas9. J Gen Virol.

[CR30] Liu X, Zhang Y, Cheng C (2017). CRISPR–Cas9-mediated multiplex gene editing in CAR-T cells. Cell Res.

[CR31] MacLeod DT, Antony J, Martin AJ (2017). Integration of a CD19 CAR into the TCR alpha chain locus streamlines production of allogeneic gene-edited CAR T cells. Mol Ther.

[CR32] Makarova KS, Haft DH, Barrangou R (2011). Evolution and classification of the CRISPR–Cas systems. Nat Rev Microbiol.

[CR33] Mali P, Yang L, Esvelt KM (2013). RNA-guided human genome engineering via Cas9. Science.

[CR34] Mandal PK, Ferreira LM, Collins R (2014). Efficient ablation of genes in human hematopoietic stem and effector cells using CRISPR/Cas9. Cell Stem Cell.

[CR35] Martins-Rocha M, Cavalheiro GM, Matos-Rodrigues GE (2015). From gene targeting to genome editing: transgenic animal applications and beyond. An Acad Bras Cienc.

[CR36] Maruyama T, Dougan SK, Truttmann MC (2015). Increasing the efficiency of precise genome editing with CRISPR–Cas9 by inhibition of nonhomologous end joinig. Nat Biotechnol.

[CR37] Menger L, Sledzinska A, Bergerhoff K (2016). TALEN-mediated inactivation of PD-1 in tumor-reactive lymphocytes promotes intratumoral T-cell persistence and rejection of established tumors. Cancer Res.

[CR38] Miller JC, Tan S, Qiao G (2011). A TALE nuclease architecture for efficient genome editing. Nat Biotechnol.

[CR39] Nemudryi AA, Valetdinova KR, Medvedev SP (2014). TALEN and CRISPR/Cas genome editing systems: tools of discovery. Acta Naturae.

[CR40] Nishimasu H, Ran FA, Hsu PD (2014). Crystal structure of Cas9 in complex with guide RNA and target DNA. Cell.

[CR41] Perez EE, Wang J, Miller JC (2008). Establishment of HIV-1 resistance in CD4^+^ T cells by genome editing using zinc-finger nucleases. Nat Biotechnol.

[CR42] Perica K, Varela JC, Oelke M (2015). Adoptive T cell immunotherapy for cancer. Rambam Maimonides Med J.

[CR43] Poirot L, Philip B, Schiffer-Mannioui C (2015). Multiplex genome-edited T-cell manufacturing platform for “off-the-shelf” adoptive T-cell immunotherapies. Cancer Res.

[CR44] Provasi E, Genovese P, Lombardo A (2012). Editing T cell specificity towards leukemia by zinc finger nucleases and lentiviral gene transfer. Nat Med.

[CR45] Qasim W, Zhan H, Samarasinghe S (2017). Molecular remission of infant B-ALL after infusion of universal TALEN gene-edited CAR T cells. Sci Transl Med.

[CR46] Ran FA, Hsu PD, Lin CY (2013). Double nicking by RNA-guided CRISPR Cas9 for enhanced genome editing specificity. Cell.

[CR47] Schumann K, Lin S, Boyer E (2015). Generation of knock-in primary human T cells using Cas9 ribonucleoproteins. Proc Natl Acad Sci USA.

[CR48] Sharma R, Anguela XM, Doyon Y (2015). In vivo genome editing of the albumin locus as a platform for protein replacement therapy. Blood.

[CR49] Shen B, Zhang W, Zhang J (2014). Efficient genome modification by CRISPR–Cas9 nickase with minimal off-target effects. Nat Methods.

[CR50] Silva G, Poirot L, Galetto R (2011). Meganucleases and other tools for targeted genome engineering: perspectives and challenges for gene therapy. Curr Gene Ther.

[CR51] Stoddard BL (2011). Homing endonucleases: from microbial genetic invaders to reagents for targeted DNA modification. Structure.

[CR52] Su S, Hu B, Shao J (2016). CRISPR–Cas9 mediated efficient PD-1 disruption on human primary T cells from cancer patients. Sci Rep.

[CR53] Su S, Zou Z, Chen F (2017). CRISPR–Cas9-mediated disruption of PD-1 on human T cells for adoptive cellular therapies of EBV positive gastric cancer. Oncoimmunology.

[CR54] Tanenbaum ME, Gilbert LA, Qi LS (2014). A protein-tagging system for signal amplification in gene expression and fluorescence imaging. Cell.

[CR55] Tebas P, Stein D, Tang WW (2014). Gene editing of CCR5 in autologous CD4 T cells of persons infected with HIV. N Engl J Med.

[CR56] Terns RM, Terns MP (2013). The RNA- and DNA-targeting CRISPR–Cas immune systems of *Pyrococcus furiosus*. Biochem Soc Trans.

[CR57] Tsai SQ, Wyvekens N, Khayter C (2014). Dimeric CRISPR RNA-guided nucleases for highly specific genome editing. Nat Biotechnol.

[CR58] Turtle CJ, Hanafi LA, Berger C (2016). Immunotherapy of non-Hodgkin lymphoma with a defined ratio of CD8^+^ and CD4^+^ CD19-specific chimeric antigen receptor-modified T cells. Sci Transl Med.

[CR59] van Loenen MM, de Boer R, Amir AL (2010). Mixed T cell receptor dimers harbor potentially harmful neoreactivity. Proc Natl Acad Sci USA.

[CR60] van der Ploeg JR (2009). Analysis of CRISPR in *Streptococcus mutans* suggests frequent occurrence of acquired immunity against infection by M102-like bacteriophages. Microbiology.

[CR61] Wang J, DeClercq JJ, Hayward SB (2016). Highly efficient homology-driven genome editing in human T cells by combining zinc-finger nuclease mRNA and AAV6 donor delivery. Nucleic Acids Res.

[CR62] Yin H, Song CQ, Dorkin JR (2016). Therapeutic genome editing by combined viral and non-viral delivery of CRISPR system components in vivo. Nat Biotechnol.

[CR63] Zaslavskiy M, Bertonati C, Duchateau P (2014). Efficient design of meganucleases using a machine learning approach. BMC Bioinform.

